# Hippo signalling as a nexus in host–virus interactions

**DOI:** 10.1371/journal.ppat.1014316

**Published:** 2026-06-11

**Authors:** Aida Bahrami, Cheryl Walter, Amin Ardestani

**Affiliations:** 1 Institute‌‌ of Biochemistry and Biophysics, University of Tehran, Tehran, Iran; 2 Centre for Biomedicine, Hull York Medical School (HYMS), University of Hull, Hull, United Kingdom; Washington University School of Medicine in Saint Louis: Washington University in St Louis School of Medicine, UNITED STATES OF AMERICA

## Abstract

The Hippo signalling pathway has emerged as an important regulator of host–virus interactions, linking antiviral immunity, viral replication, inflammation, and tissue remodelling. Recent studies show that upstream Hippo kinases such as MST1/2 and LATS1/2 often support antiviral responses, whereas YAP and TAZ can suppress innate immune signalling and may be exploited by viruses to promote infection. However, these effects are highly context dependent, varying according to viral species, cell type, infection stage, interferon signalling, inflammatory cues, and tissue damage. This short review summarises current evidence for Hippo-virus crosstalk and highlights how this pathway may shape both acute antiviral defence and longer-term pathological remodelling. We also discuss therapeutic opportunities and challenges, emphasising that targeting Hippo signalling requires caution because of its dual roles in antiviral immunity and tissue repair.

## Introduction

Innate‌‌ immune signalling represents the first line of defence against viral infection, relying on pattern-recognition receptors (PRRs) such as Toll-like receptors (TLRs), RIG-I-like receptors, and the cGAS-STING axis to induce interferons and inflammatory cytokines [1]. While canonical immune pathways have been extensively studied, it has become increasingly clear that developmental and metabolic signalling networks intersect with antiviral defence. The Hippo pathway is one such network. Originally characterised as a growth-suppressive cascade centred on the MST1/2 and LATS1/2 kinases, the pathway regulates the localisation and transcriptional activity of the co-activators YAP and TAZ through TEAD family transcription factors [2].

Over the past decade, Hippo components have been implicated in both innate and adaptive immunity [3,4]. Viral infection frequently alters Hippo signalling, either as part of host defence or through viral subversion. Given the well-established role of YAP/TAZ in tissue repair and regeneration, viral modulation of Hippo-YAP signalling likely reflects a trade-off between effective antiviral defence and preservation of regenerative capacity in infected tissues. Recent studies now suggest that this trade-off is not only driven by viral modulation of Hippo components, but also by interferon-mediated regulation of the pathway, which can simultaneously enhance antiviral signalling and restrain YAP-dependent tissue repair [5,6]. Together, recent studies reveal a series of key facts illustrating how Hippo signalling intersects with innate antiviral immunity, viral pathogenesis and inflammation-driven tissue remodelling across diverse viral infections.

## Fact: YAP/TAZ frequently act as intrinsic brakes on innate antiviral immunity

A recurring and well-supported observation is that YAP and TAZ function as intrinsic brakes on innate immune signalling, thereby limiting antiviral responses in multiple contexts. In resting cells, nuclear YAP/TAZ can suppress the transcription of PRRs or interferon-stimulated genes, reducing antiviral gene expression. Mechanistically, YAP/TAZ interfere with TBK1-IRF3 signalling, modulate chromatin accessibility at immune gene promoters, and repress PRR expression via TEAD-dependent transcriptional program. Genetic loss or pharmacological inhibition of YAP/TAZ frequently enhances interferon production and restricts viral replication, supporting a model in which YAP/TAZ constrain excessive inflammation but at the cost of reduced antiviral defence [7,8].

## Fact: Upstream Hippo kinases function as antiviral signalling modules

**Fig 1:**
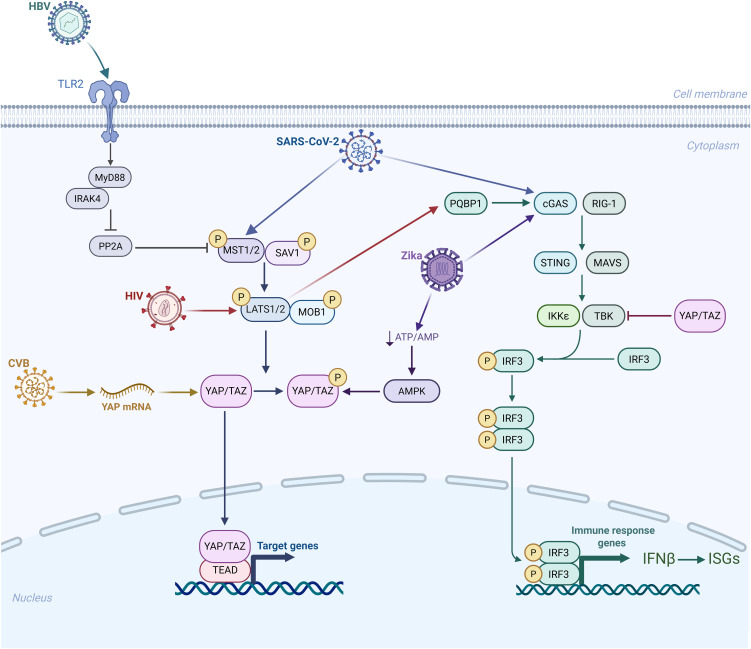
Viral modulation of Hippo-YAP signalling and its crosstalk with innate antiviral immunity. Schematic illustrating how viral infections regulate the Hippo pathway and its interaction with cytosolic antiviral sensing pathways to influence interferon responses. Hepatitis B virus (HBV) activates Toll-like receptor 2 (TLR2), signalling through MyD88 and IRAK4 to modulate PP2A and the core Hippo kinase MST1/2. Severe acute respiratory syndrome coronavirus-2 (SARS-CoV-2) activates MST1/2 and LATS1/2, while HIV also influences LATS1/2 activity and promotes crosstalk with the PQBP1-cGAS axis. Coxsackievirus B (CVB) increases YAP expression at the mRNA level. Zika virus is shown as an example of infection-associated metabolic stress, in which ATP depletion and increased AMP levels activate AMPK, linking cellular energy status to Hippo-YAP regulation and innate immune signalling. Activation of MST1/2 and LATS1/2 leads to phosphorylation of YAP/TAZ, promoting their cytoplasmic retention and regulating their transcriptional activity. When active, YAP/TAZ translocate to the nucleus and associate with TEAD transcription factors to regulate target gene expression. In parallel, viral nucleic acids are detected by cytosolic sensors including PQBP1, cGAS, and RIG-I, which signal through STING or MAVS to activate TBK1 and IKKε. These kinases phosphorylate IRF3, leading to its dimerisation, nuclear translocation, and induction of immune response genes, including interferon-β (IFNβ) and downstream interferon-stimulated genes (ISGs). Crosstalk between Hippo signalling and innate immune pathways influences the magnitude and outcome of antiviral responses. YAP/TAZ can suppress TBK1-mediated IRF3 activation, illustrating reciprocal crosstalk between Hippo signalling and innate antiviral immunity. Created in BioRender. Ardestani, A. (2026) https://BioRender.com/0dmjgn6.

In contrast to YAP/TAZ, a second key fact is that activation of upstream Hippo kinases frequently correlates with enhanced antiviral immunity. Multiple viruses, including severe acute respiratory syndrome coronavirus 2 (SARS-CoV-2), human immunodeficiency virus (HIV) and Ebola virus, induce phosphorylation of YAP at serine 127, a hallmark of MST1/2 and LATS1/2 activation and Hippo pathway ‘ON’ status (Fig 1). Under these conditions, cytoplasmic sequestration or degradation of YAP/TAZ relieves their inhibitory effects on innate immune pathways. Experimental inhibition or knockdown of MST1/2 or LATS1/2 commonly results in increased viral replication, whereas enforced Hippo kinase activity restricts infection [9–11].

Importantly, Hippo kinases also regulate immunity through non-canonical mechanisms independent of YAP/TAZ. For example, LATS2 enhances cGAS-STING signalling via interaction with PQBP1 during HIV-1 infection, promoting type I interferon production without requiring downstream YAP/TAZ transcriptional activity [12] (Fig 1). Together, these findings establish Hippo kinases as direct immune modulators rather than merely upstream regulators of YAP/TAZ.

Also, recent studies show that interferons can directly activate Hippo kinase signalling, adding an important feedback layer to Hippo-antiviral crosstalk. Zuo et al. identified LATS1 as a key transmitter of type I interferon activity: IFN-I receptor engagement activates LATS1 through a Tyk2-dependent mechanism, promoting YAP degradation and enabling full antiviral signalling via a LATS1-CDK8-STAT1 axis. Consistently, LATS1 deficiency attenuates IFN-I-induced antiviral responses in vivo [5]. A complementary study in human bronchial epithelial cells showed that type I and type III interferons activate LATS1 through JAK-dependent, MST1/2-independent signalling, thereby suppressing YAP-dependent epithelial migration and proliferation. This work further suggests that LATS1 activation requires higher interferon concentrations than STAT1 activation, providing a dose-dependent mechanism by which interferons may coordinate antiviral defence with delayed tissue repair [6]. Together, these findings position LATS1 as both an amplifier of interferon-mediated antiviral activity and a mediator of interferon-dependent restraint of tissue regeneration.

## Fact: Hippo pathway regulation during viral infection is context-dependent

A further important fact is that the impact of Hippo signalling on antiviral immunity is context dependent, with divergent outcomes observed across tissues, cell types and viruses. Interplay between Coxsackievirus A10 and Zika virus with Hippo signalling illustrates this variability. In the central nervous system, Coxsackievirus A10 infection of microglia robustly activates PRR-driven inflammatory cytokine production, while MST1/2 simultaneously suppress IRF3-dependent interferon signalling and enhance viral replication. Mechanistically, MST1/2 interact with TBK1 and IRAK1, uncoupling antiviral interferon responses from NF-κB-mediated inflammation [13].

Zika virus further exemplifies layered regulation of Hippo signalling during infection. Zika virus protein NS1 enhances NLRP3 inflammasome activity by stabilising caspase-1, promoting caspase-1-mediated cleavage of cGAS and dampening cGAS-dependent type I interferon responses to benefit viral replication [14]. In parallel, Zika virus infection engages AMPK-Hippo-TBK1 crosstalk, linking antiviral signalling to cellular energy-stress responses, particularly in neural and ocular tissues [15] (Fig 1).

**Fig 2:**
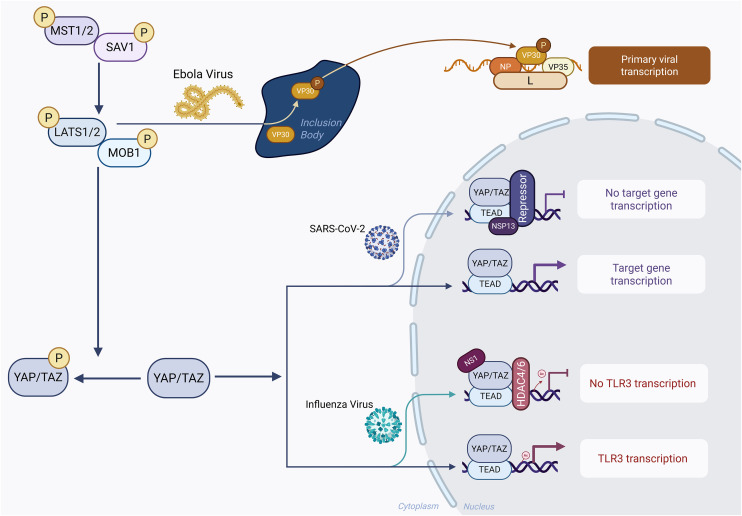
Viral manipulation of Hippo-YAP/TAZ signalling to regulate viral replication and host transcriptional responses. Schematic showing how different viruses interact with the Hippo pathway to control YAP/TAZ activity and downstream transcription. Activation of the core Hippo kinases MST1/2 and LATS1/2 and their partners SAV1 and MOB1 leads to phosphorylation of YAP/TAZ, regulating their subcellular localisation and transcriptional function. During Ebola virus infection, LATS1/2 is recruited to viral inclusion bodies, where it phosphorylates the viral protein VP30. Phosphorylated VP30 participates in the viral transcriptional complex containing NP, VP35, and the viral polymerase L, thereby regulating primary viral transcription. During SARS-CoV-2 infection, YAP/TAZ translocate to the nucleus and interact with TEAD transcription factors to regulate host gene expression. However, the viral protein helicase NSP13 promotes recruitment of transcriptional repressors to the YAP/TAZ-TEAD4 complex, suppressing target gene transcription. TEAD4 may function as a scaffold to recruit both NSP13 and YAP, and NSP13 likely inactivates the YAP/TEAD4 transcriptional complex by inducing chromatin remodelling and recruiting repressive proteins to the YAP/TEAD/NSP13 complex. During influenza A virus infection, the viral non-structural protein NS1 physically interacts with the C-terminal domain of YAP/TAZ within the YAP/TAZ–TEAD complex, enabling recruitment of histone deacetylases HDAC4/6 to the TLR3 promoter. YAP binds a putative TEAD-binding motif at this locus, and HDAC4/6-mediated reduction of histone H3 acetylation leads to chromatin remodelling and transcriptional silencing of TLR3, thereby suppressing antiviral innate immune signalling. In the absence of this repression, YAP/TAZ-TEAD promote TLR3 transcription. These examples illustrate that viruses can manipulate Hippo-YAP signalling at multiple levels, including upstream kinase activity, viral protein phosphorylation, and YAP/TEAD-dependent host transcription. Created in BioRender. Ardestani, A. (2026) https://BioRender.com/ihv7etr.

Ebola virus provides another example of context-dependent Hippo-virus interaction among RNA viruses. LATS1/2 can directly phosphorylate viral proteins to regulate Ebola virus transcription and egress, while YAP localisation influences whether viral budding is promoted or inhibited [11] (Fig 2).

This example reinforces the broader point that Hippo pathway components cannot be universally classified as proviral or antiviral, but instead act in a virus- and context-specific manner.

## Fact: Viruses actively hijack YAP/TAZ and the YAP-TEAD transcriptional complex

Another key fact emerging from recent work is that multiple viruses actively exploit YAP/TAZ and the YAP-TEAD transcriptional complex to suppress host antiviral responses. Influenza A virus NS1 physically interacts with YAP/TAZ, promoting their nuclear localisation and enabling repression of TLR3 expression through TEAD-dependent chromatin remodelling [16] (Fig 2). This hijacking strategy dampens pro-inflammatory and antiviral cytokine production, facilitating viral replication and pathogenesis.

Beyond YAP/TAZ themselves, viruses can directly target the YAP-TEAD transcriptional complex. During SARS-CoV-2 infection, transcriptomic analyses of infected human cells and tissues reveal reduced expression of canonical YAP target genes. Mechanistically, the viral helicase NSP13 binds TEAD4 and associates with the YAP-TEAD complex, suppressing transcriptional output independently of upstream LATS1/2 activity [17] (Fig 2). This provides a molecular explanation for how Hippo pathway activation signatures can coexist with reduced YAP/TEAD-driven gene expression during infection.

## Case study: Multilayered Hippo pathway regulation during SARS-CoV-2 infection

Studies of SARS-CoV-2 provide a clear example of how multiple layers of Hippo pathway regulation can operate simultaneously during viral infection. Infection activates Hippo kinases in lung and cardiac models, and genetic or pharmacological inhibition of MST1/2 or LATS1 enhances viral replication, supporting an antiviral role for Hippo kinase signalling [9]. Conversely, SARS-CoV-2 can suppress YAP/TEAD transcriptional activity through viral protein-mediated targeting of the TEAD complex, as discussed above. Thus, SARS-CoV-2 infection may initiate a combination of host-driven Hippo activation and direct viral mechanisms that reshape YAP/TEAD-dependent transcription. The relative contribution of these layers is likely determined by timing, cell type, viral burden, and inflammatory context.

## Fact: DNA viruses also intersect with Hippo signalling through diverse mechanisms

Beyond RNA viruses, accumulating evidence indicates that DNA viruses also intersect with Hippo signalling at multiple regulatory levels. Hepatitis B virus activates TLR2-dependent signalling that converges on both NF-κB and Hippo pathways, with YAP contributing to negative feedback regulation of inflammatory responses through control of IκBα expression [18] (Fig 1). Human cytomegalovirus, in contrast, is inhibited by YAP, which suppresses STING expression and impairs nuclear transport of the viral genome, highlighting antiviral roles for YAP in specific contexts [19].

These examples indicate that, even among DNA viruses, Hippo pathway components can have context-dependent effects on viral replication and host antiviral responses.

## Fact: Hippo-YAP signalling links viral infection and antiviral inflammation to tissue remodelling and chronic disease

Beyond acute antiviral responses, a key emerging fact is that Hippo-YAP signalling couples viral infection and sustained antiviral inflammation to long-term tissue remodelling and chronic disease pathology. During persistent HIV-1 infection, sustained YAP activation via a non-canonical LPA-LPAR1-PI3K-AKT axis drives progressive liver fibrosis, independent of direct effects on viral replication [10]. These findings highlight how chronic viral infection can rewire Hippo signalling to promote fibrotic pathology. However, this is not universal across all viral infections. In HBV infection, increased matrix stiffness activates YAP in hepatocytes and suppresses viral transcription and antigen production, consistent with a non-cytolytic antiviral restriction mechanism. Notably, stiffness increases observed during HBV infection can be transient and independent of established fibrosis, underscoring the context-dependent consequences of YAP activation, which may contribute either to antiviral defence or pathological tissue remodelling depending on the infection setting [20].

Hippo-YAP signalling is also implicated in enteroviral infections associated with the initiation of type 1 diabetes (T1D) pathogenesis [21]. We have recently shown that Coxsackievirus B (CVB) infection induces inflammatory signalling that intersects with Hippo pathway components, influencing both viral replication and host immune responses in pancreatic tissue. YAP expression closely tracks viral burden, with frequent colocalisation of YAP and CVB RNA observed within infected human pancreatic cells and significantly greater viral clustering in autopsy samples from T1D donors compared with non-diabetic donors. Functional studies establish a causal role for YAP in viral propagation: CVB infection induces both YAP mRNA and protein expression (Fig 1) and YAP overexpression enhances enteroviral replication, islet inflammation, and insulin-producing β-cell apoptosis, whereas YAP inhibition blocks viral replication in both primary and immortalised pancreatic cells [21]. These findings position YAP as a regulator of virus-induced β-cell stress and inflammation, suggesting that dysregulated Hippo-YAP signalling may contribute to β-cell vulnerability, and virus-triggered autoimmunity in T1D.

Beyond direct control of viral replication, Hippo-YAP signalling also links antiviral inflammation to pathological tissue remodelling in other organs. During IFN-γ-driven alveolar remodelling in severe viral pneumonia as a result of Influenza A virus infection, YAP is activated through a non-canonical FAK/Src pathway, promoting the expansion of dysplastic epithelial cells that impair normal alveolar regeneration. Inhibition of YAP reverses this maladaptive remodelling, implicating Hippo-YAP signalling as a mediator of tissue pathology downstream of sustained antiviral inflammation [22].

It is also important to distinguish direct viral manipulation of Hippo components from indirect changes in Hippo pathway activity that arise as a consequence of infection. Viral proteins can directly target YAP/TAZ, or upstream Hippo kinases; however, infection-associated interferon production, inflammatory cytokines, epithelial injury, altered tissue mechanics, and regenerative responses may also activate LATS1/2 or YAP independently of direct viral targeting. Thus, Hippo pathway changes observed during infection may reflect a combination of virus-intrinsic mechanisms and host-driven responses to tissue damage and antiviral inflammation.

## Therapeutic implications and challenges

From an evolutionary perspective, the recurrent targeting of Hippo signalling by diverse viral families suggests that this pathway originally evolved as a component of the host antiviral defence. The emergence of YAP as a frequently co-opted proviral factor likely reflects a secondary adaptation, whereby viruses exploit an existing immune-suppressive function most notably YAP-mediated inhibition of IRF3 rather than evolving entirely novel antagonists. This evolutionary logic reinforces YAP as a convergent vulnerability across viral species. The expanding role of Hippo-YAP signalling in viral infection has therefore prompted interest in pharmacological targeting of this pathway. Verteporfin, a clinically approved YAP-TEAD inhibitor, exhibits antiviral activity against multiple RNA virus families, including SARS-CoV-2 and several established and emerging arboviruses [23]. However, verteporfin and related compounds may exert pleiotropic effects beyond YAP-TEAD inhibition, and broad suppression of YAP activity carries risks given its roles in tissue repair and regeneration.

Additional strategies aimed at modulating YAP activity include statins, which retain YAP/TAZ in the cytoplasm [24], and approaches that accelerate YAP degradation through ARRDC3 activation, lysosomal pathways, or emerging PROTAC-based technologies [25,26]. Targeting upstream inflammatory or metabolic inputs that converge on YAP activation, including LPA and TLR signalling [10,16], represents another therapeutic avenue. However, viruses also exploit upstream Hippo kinases, creating both opportunities and cautions for intervention. Loss or inhibition of MST1/2 or LATS1/2 enhances viral replication in several contexts, including SARS-CoV-2 infection [9], whereas human MST1 deficiency is associated with immunodeficiency and increased susceptibility to viral infection [27,28]. Conversely, LATS1/2 inhibition suppresses Ebola virus transcription and virion production despite concomitant YAP activation, revealing virus-specific therapeutic windows [11].

Together, these findings highlight Hippo-YAP signalling as a promising but complex therapeutic axis. Therapeutic targeting of Hippo signalling should therefore be approached with caution, as excessive suppression or activation of this pathway may have unintended consequences for tissue repair, pathological remodelling, and viral replication. The therapeutic window is likely to depend on the viral species, affected tissue, timing of infection, and whether YAP/TAZ or upstream MST/LATS kinases are the dominant drivers of pathology. Defining these parameters will be essential for harnessing Hippo signalling as a safe and effective target in antiviral and anti-fibrotic therapies.

## Conclusions and future perspectives

Collectively, current evidence positions the Hippo pathway as a central and context-sensitive regulator of antiviral immunity, viral replication, and tissue remodelling. Rather than operating as a simple on-off switch, Hippo signalling integrates host defence mechanisms with viral countermeasures at multiple regulatory levels. Future studies should prioritise temporal and cell-type-specific analyses, clarify non-canonical Hippo signalling in immunity, and define how viral proteins selectively rewire YAP/TEAD transcriptional program. A deeper mechanistic understanding will be essential for safely exploiting Hippo-YAP signalling as a therapeutic target in viral disease, chronic inflammation, and virus-triggered autoimmunity.
